# The Assertive Brain: Anterior Cingulate Phosphocreatine plus Creatine Levels Correlate With Self-Directedness in Healthy Adolescents

**DOI:** 10.3389/fpsyt.2019.00763

**Published:** 2019-11-05

**Authors:** Letizia Squarcina, Giuseppe Delvecchio, Maria Nobile, Maddalena Mauri, Domenico Madonna, Carolina Bonivento, Marco Garzitto, Sara Piccin, Massimo Molteni, Barbara Tomasino, Cinzia Bressi, Franco Fabbro, Jeffrey A. Stanley, Paolo Brambilla

**Affiliations:** ^1^Department of Neurosciences and Mental Health, Foundation IRCCS Ca’ Granda Ospedale Maggiore Policlinico, Milan, Italy; ^2^Department of Pathophysiology and Transplantation, University of Milan, Milan, Italy; ^3^Child Psychopathology Unit, Scientific Institute, IRCCS Eugenio Medea, Bosisio Parini, Italy; ^4^School of Medicine and Surgery, University of Milano-Bicocca, Milan, Italy; ^5^Scientific Institute, IRCCS Eugenio Medea, San Vito al Tagliamento, Italy; ^6^Department of Psychiatry and Behavioral Neurosciences, School of Medicine, Wayne State University, Detroit, MI, United States

**Keywords:** magnetic resonance spectroscopy, temperament character inventory, adolescence, brain biochemistry, brain metabolism

## Abstract

Despite various advances in the study of the neurobiological underpinnings of personality traits, the specific neural correlates associated with character and temperament traits are not yet fully understood. Therefore, this study aims to fill this gap by exploring the biochemical basis of personality, which is explored with the temperament and character inventory (TCI), during brain development in a sample of adolescents. Twenty-six healthy adolescents (aged between 13 and 21 years; 17 males and 9 females) with behavioral and emotional problems underwent a TCI evaluation and a 3T single-voxel proton magnetic resonance spectroscopy (^1^H MRS) acquisition of the anterior cingulate cortex (ACC). Absolute metabolite levels were estimated using LCModel: significant correlations between metabolite levels and selective TCI scales were identified. Specifically, phosphocreatine plus creatine (PCr+Cre) significantly correlated with self-directedness, positively, and with a self-transcendence (ST), negatively, while glycerophosphocholine plus phosphocholine (GPC+PC) and myo-inositol negatively correlated with ST. To the best of our knowledge, this is the first study reporting associations of brain metabolites with personality traits in adolescents. Therefore, our results represent a step forward for personality neuroscience within the study of biochemical systems and brain structures.

## Introduction

The genetic and neural underpinnings of personality traits have gained increasing interest within the scientific community. Indeed, in the last decades, many studies have started to assess human personality from a scientific point of view, with the final aim of disentangling the neural basis of personality dimensions.

Interestingly, the majority of neuroimaging and behavioral studies employed the temperament and character inventory (TCI) (1) with the aim of investigating personality traits in normal subjects or in patients. The TCI is a well known personality inventory developed by Robert Cloninger, which models personality using seven psychobiological factors (1). It is composed of four temperament and three character scales. Temperament dimensions refer to the way each individual behaviorally responds to a specific class of stimuli, while character dimensions refer to self-concepts and inter-individual differences in goals and values, which may be associated to the functioning of higher cognitive systems. For details on the definition of each scale, please refer to a previous work of our group (2). Notably, twin studies reported that genetic factors have significant effects on temperament and on character dimensions, where the heritability does not show strong differences (3–5).

With regards to behavioral studies, evidence from our research group reported that selective temperament and character dimensions were associated with selective impairments in decision-making, during adolescence (6), magic ideations in twins (7) and proved to be useful for describing the development of personality in childhood (2). Additionally, a recent study carried out by Crescentini et al. (8) also reported that specific temperament and character traits might have protective effects on well being and psychosocial adjustment or explain emotional–behavioral difficulties in adolescents. Specifically, the study carried out by Brambilla et al. (7) found significant correlations between magical ideation and specific personality traits such as novelty seeking, cooperativeness, self-directedness, and self-transcendence in a sample of adult twins, mostly explained by genetic factors. In particular, self-directedness, a major proxy of psychological consciousness and confidence, and self-transcendence, an indication for spirituality and mysticism, are negatively and positively, respectively, associated with magical ideation.

From a neurobiological perspective, although the identification of specific brain deficits associated with personality traits is of great interest, the neurobiological bases of character and temperament dimensions are not yet fully understood. However, the available evidence reported some interesting results. Indeed, some studies highlighted the link between personality characteristics and connectivity areas in the brain (9) and white matter integrity (10, 11). Moreover, the review and meta-analysis carried out by Mincic (12) showed that the personality trait of negative emotionality was associated with selective deficits in brain regions within the cortico-limbic system, ultimately implying alterations in information communication and processing. Authors reported reduced gray matter volumes in the left medial orbitofrontal gyrus and rostral anterior cingulate cortex and increased volumes in the left amygdala and anterior parahippocampal gyrus in individuals who have predominant negative traits regarding emotions. This study's results further confirm the presence of morphological alterations associated with negative personality traits, as also reported by previous studies (13, 14).

Interestingly, the paucity of neuroimaging studies on personality traits is also present in regards to proton magnetic resonance spectroscopy (^1^H MRS) investigations. ^1^H MRS is the only technique that can access *in vivo* metabolite levels including N-acetylaspartate (NAA), phosphocreatine plus creatine (PCr+Cre), glycerophosphocholine plus phosphocholine (GPC+PC), and myo-inositol in localized brain areas (15, 16). The available *in vivo* ^1^H MRS evidence on personality traits suggested that the individual variation in absolute brain metabolites levels may relate to specific aspects of personality functioning in healthy individuals. For example, lower PCr+Cre levels in the right precuneus were associated with agreeableness and extraversion, indicating a possible lower production of high-energy phosphate, PCr, correlating with these traits (17). Similarly, Kim et al. (18) explored the association between functional/structural alteration of the anterior cingulate cortex and harm avoidance traits. The authors showed that harm avoidance scores correlated negatively with glutamate concentrations and positively with GABA concentrations in anterior cingulate cortex, ultimately suggesting that glutamate and GABA concentrations in anterior cingulate cortex could underline the HA temperament trait.

In this context, this study aims at exploring, for the first time to the best of our knowledge, the biochemical basis of personality during brain development in a sample of adolescents with the final goal of teasing apart the biochemical system associated with personality traits. We hypothesized an association of PCr+Cre levels from the anterior cingulate cortex with selective personality traits in healthy individuals. This hypothesis derives by the evidence reported by previous studies in both healthy (19) and depressed (20) adolescents, which showed the key role of creatine’s modulation in brain energy metabolism. Furthermore, since Kondo et al. (20) also found that creatine levels within the frontal lobe were inversely associated with depressive symptoms, we expect that PCr+Cre levels will be more likely be associated with SD, a personality trait consistently found associated with depression (21, 22).

## Materials and Methods

### Participants

Twenty-six subjects (17 males; 9 females) took part to the study. The participants were selected within a cohort of adolescents, aged between 13 and 21 years (mean age ± 1 SD: 16.9 ± 1.7 years old), referred to the Istituto di Ricovero e Cura a Carattere Scientifico (IRCCS) “E.Medea” (Italy) between 2003 and 2008 because of behavioral or emotional problems such as anxiety and attentional deficit (see socio-demographic and clinical details in **Table 1**). Within the main cohort, we selected those subjects who did not meet criteria for a ICD-10 and DSM-IV diagnoses at the time of the study according to the Development and Well-Being Assessment (DAWBA; 23). Participants with reports of an IQ lower than 70 on their medical record, or diagnosed with a pervasive developmental disorder, severe hypoacusia or hypovision, severe linguistic comprehension deficit, central nervous system lesion, neurological condition, or a genetic syndrome, were excluded.

**Table 1:** Socio-demographic and clinical variables of our sample.

N = 26 (9 females)	Minimum	Maximum	Mean	Std. deviation
**Age**	14.4	20.8	16.9	1.7
**SES**	20.0	80.0	50.5	14.6
**Estimated IQ (based on vocabulary and block design)**	82.5	127.5	101.7	10.9
**CBCL-Int**	34.0	73.0	55.1	9.9
**CBCL-Ext**	34.0	66.0	51.7	8.3
**TCI-SD**	6.0	24.0	17.1	4.9
**TCI-ST**	0.0	8.0	3.1	2.0
**TCI-CO**	9.0	24.0	19.0	3.4
**TCI-NS**	2.0	17.0	10.1	4.1
**TCI-HA**	1.0	17.0	8.8	4.3
**TCI-RD**	3.0	13.0	8.6	2.9
**TCI-P**	0.0	5.0	2.5	1.4

CBCL, child behavior checklist; SES, socio-economic status; estimated IQ, IQ estimated from the Vocabulary and Block Design subtests of the Wechsler Intelligence Scale for Children (WISC—13–15 years) and Wechsler Adult Intelligence Scale (WAIS—16 and older); TCI, Temperament and Character Inventory; Int, internalizing problems index; Ext, externalizing problems index; SD, self-directedness; ST, self-transcendence; CO, cooperativeness; NS, novelty seeking; HA, harm avoidance; RD, reward dependency; P, persistence. The means of the weighted scores were converted into standard scores.

Also, the children’s parents filled the Child Behavior Checklist (CBCL 6-18) (24), which evaluates the behavior (see below for a full description—**Table 1** reports the results).

All children and their parents were Italian native speakers or were fluent in Italian. The study was approved by the Ethical committee of the IRCCS “E. Medea.” All parents gave written informed consent.

### Psycho-Cognitive Measures

Participants were assessed to exclude behavioral, emotional, psychiatric, or neurological disorders with the DAWBA (23). The DAWBA parent interview and the DAWBA interview for young people were administered to all participants. DAWBA is a tool allowing for a structured diagnosis according to both DSM-IV (25) and ICD-10 (26). The participants IQ was tested with the vocabulary and block design subtests of the Wechsler Intelligence Scale for Children (WISC—13–15 years) and Wechsler Adult Intelligence Scale (WAIS—16 and older). For each participant, the IQ was estimated converting the mean of the weighted scores into standard scores. Parental socio-economic status was also estimated.

Subjects were assessed with the Child Behavior Checklist (CBCL 6-18) (24), a questionnaire assessing social competences and behavioral problems in children from 6 to 18 years of age. The questionnaire is filled by one or both parents who evaluate the child behavior with reference to a period encompassing the past 6 months. The CBCL 6-18 is composed by eight syndrome scales based on factor analysis: anxious/depressed, withdrawn/depressed, somatic complaints, social problems, thought problems, attention problems, rule-breaking behavior, aggressive behavior, and six DSM-oriented scales: affective problems, anxiety problems, somatic problems, attention deficit/hyperactivity, and oppositional defiant behavior (24).

Each item consists in a statement describing a target behavior. The parents must indicate if a statement apply completely (score = 2), partially (score = 1), or does not apply (score = 0) to their children. For each syndrome and DSM-oriented scale, scores are calculated as the sum of the scores of each item in that scale. The internalizing problems index is obtained as the sum of the scores of the anxious/depressed, withdrawn/depressed, and somatic complaint scales. The externalizing problems index is given by the sum of the rule-breaking behavior and aggressive behavior scales. A total problem index is given by the sum of all scores and the scores assigned to the items of an additional scale measuring “other problems” (i.e., a scale whose items do not refer to any specific syndrome). The raw scores are then converted into T standard scores according to the child’s age and gender. We used the internalizing and externalizing problems indexes as covariates for the regression analyses. In the present study, we administered the CBCL 6-18 Italian independent back translation authorized and approved by T. Achenbach.

The TCI (1) is a self-report questionnaire measuring the seven dimensions of temperament and character postulated according to the psychobiologic model of personality (1). The temperament scales are as follows. Each of the dimensions of the TCI, except persistence, is computed as the sum of scores on three to five subscales which measure correlated traits. Here, we used a shorter version with 125 items, previously validated on a large cohort of healthy individuals by our research group (27). The questions can be clustered into four sub-scales tapping each temperament component: novelty seeking (20 items), harm avoidance (20 items), reward dependence (15 items), persistence (5 items), and into three sub-scales tapping each character component: self-directedness (25 items), cooperativeness (25 items), and self-transcendence (15 items).

### Magnetic Resonance Imaging (MRI) and Spectroscopy (MRS) Acquisition

**Figure 1:**
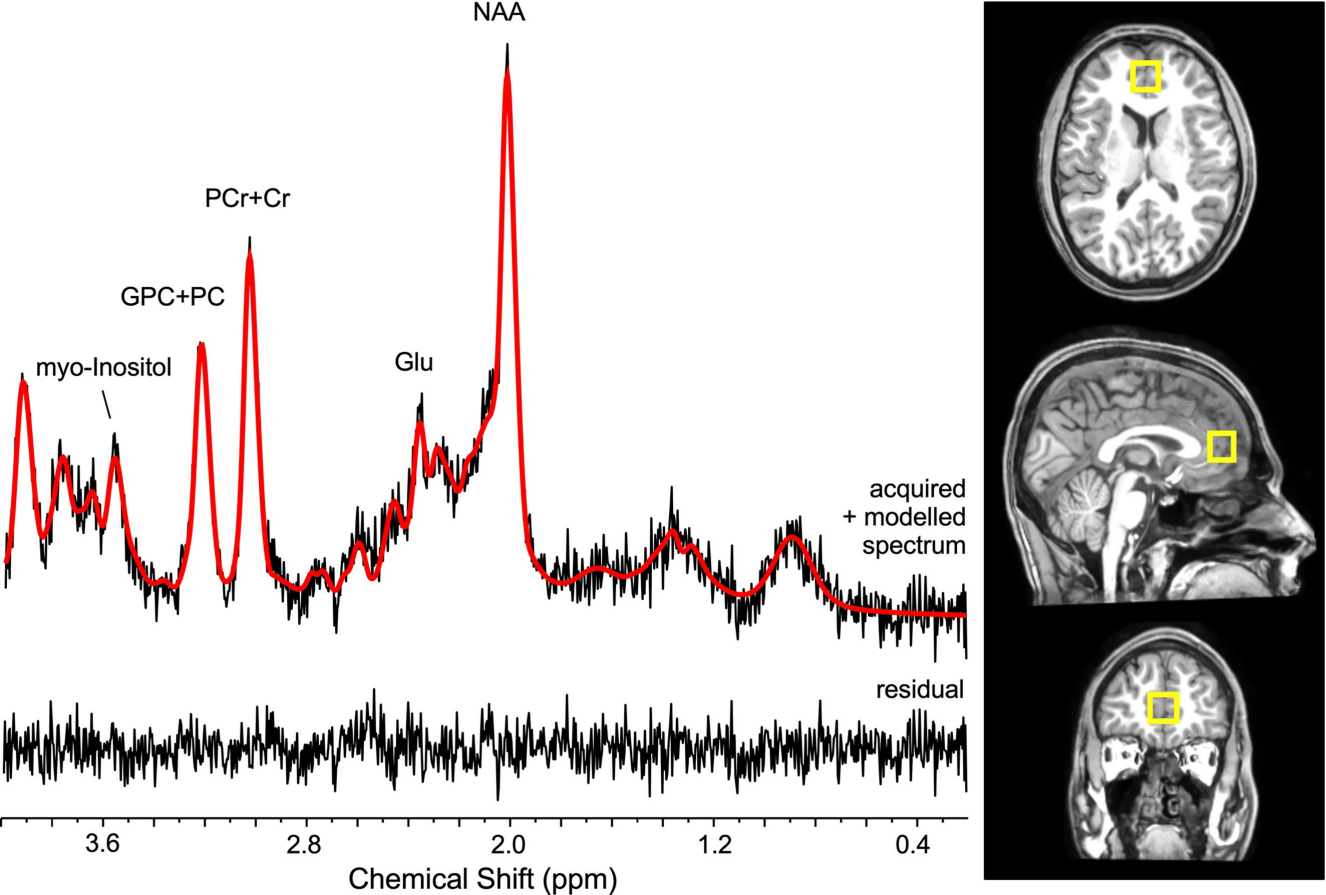
Sample spectrum (left) and voxel positioning in the anterior cingulate cortex (right).

Single-voxel *in vivo* ^1^H MRS spectra were acquired using the point-resolved spectroscopy (PRESS) sequence on a 3T whole-body MR system (Philips Achieva, Philips, the Netherlands; TR = 3,000 ms, TE = 36 ms, voxel sixe 17 x 17 x 17 mm = 4.91 mm^3^, 2,048 complex data points, spectral bandwidth of 2,000 Hz, 128 water suppressed, and 2 water unsuppressed averages). The ^1^H MRS voxel was positioned in the anterior cingulate cortex as depicted in **Figure 1**. 3D T1-weighted images (190 slices, TR = 8.2 ms, TE = 3.75 ms, flip angle = 8°, FOV = 240x240 mm, pixel dimension = 1x1x1 mm^3^) were also acquired. The proportions of tissue content of gray matter, white matter, and cerebro-spinal fluid within the localized voxels were estimated using FSL and FreeSurfer (28; http://surfer.nmr.mgh.harvard.edu/).

Absolute metabolite levels were estimated using the linear combination model [LCModel, version 6.3-1 (29)] software with a simulated basis set, the unsuppressed water signal, and by incorporating the appropriate correction factors (T1 and T2 relations) (Gasparovic et al. (30)). The quantified metabolites included PCr+Cre, GPC+PC, myo-inositol, glutamate, and NAA and expressed in absolute levels with institutional units.

### Correlations

Preliminary Pearson correlations and scatter plots were run in order to identify associations between metabolites levels and the TCI measures, to highlighting the direction of the relation between the predictors and the dependent variables. Correlations between the different measures of the metabolite levels (i.e., NAA, PCr+Cre, GPC+PC, myo-inositol, and glutamate), which were the variable that were used in the regressions as predictors, were computed for verifying the collinearity between predictors.

### Regression Analysis

Analyses were conducted using SPSS version 23 (SPSS Inc, Chicago). Four independent linear regression models were calculated, using block entry method (SPSS default), with raw scores at each TCI-125 subscale as dependent variable. The metabolite levels of NAA, PCr+Cre, GPC+PC, myo-inositol, and glutamate were entered as independent variables, one at a time. So, individual models were run for each metabolite at a time. All the regressions models analyzed also included as covariates age and gender. Moreover, CBCL internalizing and externalizing indexes (T scores) were entered as additional covariates. This was done in order to account for the correlation that in some cases existed between TCI scores and those covariates (see **Table S1** in **Supplementary Materials**).

The alpha level (significance level) = 0.05 was adjusted in order to account for the number of model dividing it by the number of models N = 6. Only the p-values less or equal to 0.05/6 = 0.01 allowed for the rejection of the null hypothesis.

## Results

### Descriptive Statistics

Subjects had a total IQ of 101.73 ± 10.88 (range 82.5–127.5) estimated with WISC or WAIS depending on age. The parental socio-economical status (SES) ranged between 20 and 80 (mean = 50.51; standard deviation = 14.55). The CBCL and TCI scores are reported in **Table 1**. **Table 2** reports the concentration levels of each metabolite. The number of normal, clinical, and subclinical subjects is reported in **Table S2**.

**Table 2:** Metabolites levels measured using ^1^H MRS.

	Mean (I.U.)	StDev (I.U.)	Min (I.U.)	Max (I.U.)
**PCr+Cre**	9.4	0.7	8.0	10.6
**GPC+PC**	2.1	0.3	1.6	2.9
**Myo-inositol**	6.6	1.0	4.9	8.6
**Glutamate**	14.3	1.0	12.2	16.1
**NAA**	10.4	0.9	8.8	12.1

StDev, 1 standard deviation; Min, minimum value; Max, maximum value. PCr+Cre, phosphocreatine plus creatine; GPC+PC, glycerophosphocholine plus phosphocholine; NAA, N-acetylaspartate; I.U., institutional units.

### Regression and Correlation Analyses

The analysis showed significant correlations between i) NAA and PCr+Cr (r = 0.38, p = 0.05), ii) NAA and GPC+PC (r = 0.49, p = 0.01), iii) PCr+Cr and GPC+PC (r = 0.78, p < 0.001), iv) PCr+Cr and MYO (r= 0.60, p < 0.001), v) PCr+Cr and Glu (r = 0.60, p < 0.001), and vi) GPC+PC and MYO (r = 0.63, p < 0.001). Since most of the metabolites’ concentrations correlated, we decided to perform separate analyses using one metabolite at a time as predictor.

**Figure 2:**
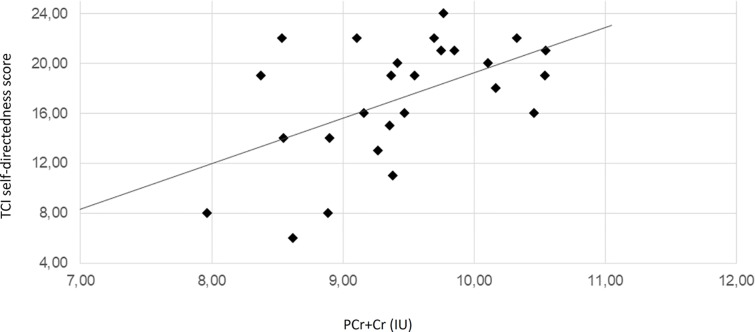
Scatter plot depicting the relationship of PCr+Cre level and TCI self-directedness scores. The solid line represents the linear regression line. The correlation coefficient r is 0.52 (Pearson correlation, p = 0.006). IU, institutional units.

**Figure 3:**
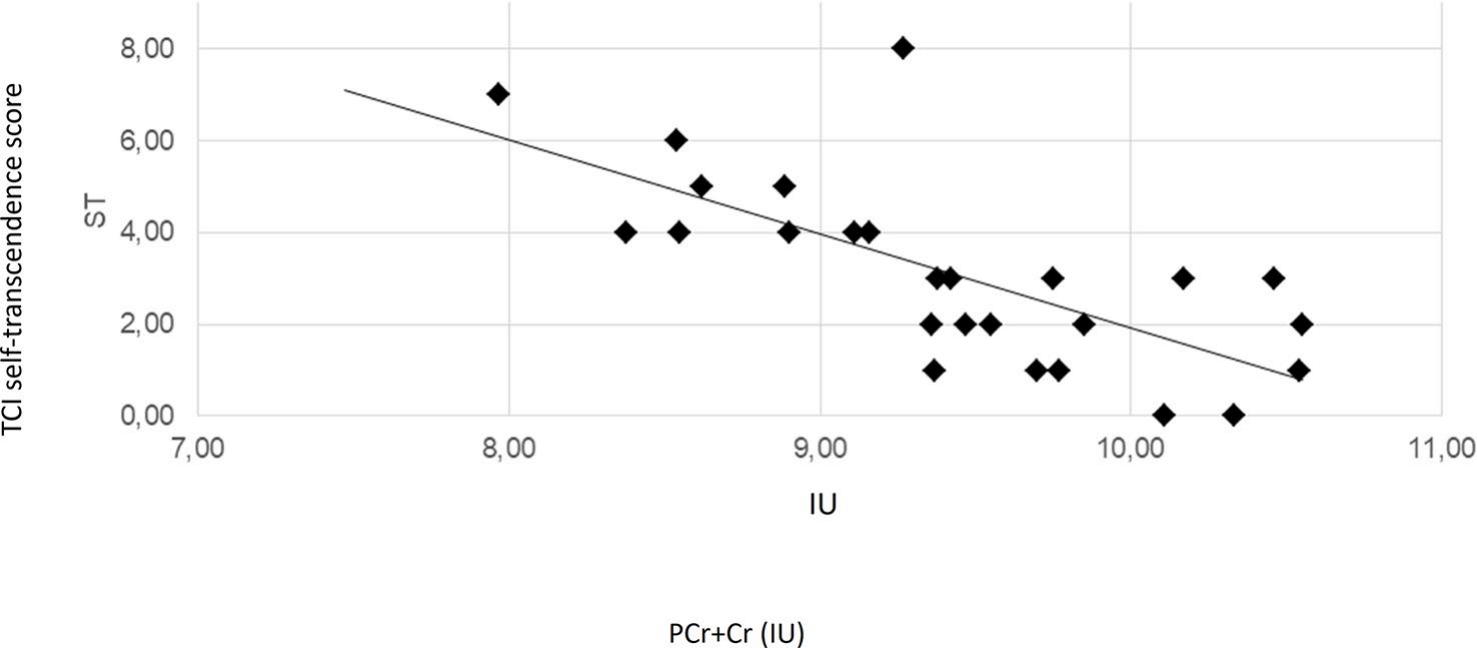
Scatter plot depicting the relationship of PCr+Cre concentration and TCI self-transcendence scores. The solid line represents the linear regression line. The correlation coefficient r is **−**0.71 (Pearson correlation, p < 0.001). IU, institutional units.

The regression analyses revealed that the concentration of PCr + Cr predicted significantly the outcome at the TCI_SD. Moreover, PCr + CR could predict the outcome at the TCI_ST. Additionally, TCI_ST was also predicted by the concentration levels of GPC + PC and myo-inositol. The ACC PCr+Cre levels significantly predicted positively the SD and negatively the ST. The ST scores were also significantly predicted by the levels of GPC+PC and myo-inositol and, in both cases, the relation was inverse. Scatter plots for these quantities are represented in **Figure 2**, **Figure 3**, **Supplementary Figure 1**, and **Supplementary Figure 2**. Results from regression analysis are summarized in **Table 3**.

**Table 3:** Results of linear regression, performed using the forced block entry method.

	Character									Temperament											
	Self-directedness			Self-transcendence			Cooperativeness			Novelty seeking			Harm Avoidance			Reward Dependence			Persistence		
	R2 (Beta)	F	p	R2 (Beta)	F	p	R2 (Beta)	F	p	R2 (Beta)	F	p	R2 (Beta)	F	p	R2 (Beta)	F	p	R2 (Beta)	F	p
PCr+Cre	**0.61 (2.99)**	**8.9**	**0.01**	**0.58 (−1.96)**	**20.4**	**<0.001**	0.13 (0.69)	0.4	0.5	0.31 (**−**0.28)	0.07	0.8	0.33 (0.98)	0.7	0.4	0.18 (0.64)	0.5	0.5	0.11 (**−**0.11)	0.1	0.8
GPC+PC	0.50 (4, 22)	2.8	0.1	**0.40 (−3.40)**	**8.7**	**0.01**	0.13 (1.53)	0.4	0.5	0.31 (**−**0.74)	0.1	0.7	0.31 (**−**0.27)	0.0	0.9	0.16 (**−**0.32)	0.0	0.9	0.12 (**−**0.37)	0.1	0.7
Myo-inositol	0.47 (1.05)	1.5	0.2	**0.45 (−1.20)**	**11.0**	**<0.001**	0.13 (**−**0.39)	0.2	0.6	0.30(**−**0.17)	0.05	0.8	0.35 (0.90)	1.2	0.3	0.17 (0.32)	0.3	0.6	0.13 (0.22)	0.5	0.5
Glutamate	0.58 (**−**0.14)	6.9	0.02	0.29 (**−**0.84)	4.1	0.1	0.12 (0.34)	0.2	0.7	0.32 (0.63)	0.6	0.4	0.32 (0.39)	0.2	0.7	0.16 (0.07)	0.01	0.9	0.11 (**−**0.00)	0.0	1.0
NAA	0.54 (1.75)	4.7	0.04	0.22 (**−**0.62)	2.0	0.1	0.12 (0.31)	0.1	0.7	0.37 (**−**1–19)	2.3	0.1	0.31 (0.24)	0.1	0.8	0.17 (**−**0.41)	0.4	0.5	0.11 (**−**0.05)	0.0	0.9

Bold values are statistically significant (p = 0.01 after Bonferroni correction). PCr+Cre, phosphocreatine plus creatine; GPC+PC, glycerophosphocholine plus phosphocholine; NAA, N-acetylaspartate.

## Discussion

The investigation of personality traits in association with regional brain biochemistry, as explored in this study, might allow building prediction models, which identify specific biomarkers associated with inter-individual differences in personality traits.

In this study, we report significant associations of PCr+Cre, GPC+PC, and myo-inositol levels in the anterior cingulate cortex with specific and opposite personality traits. Specifically, PCr+Cre significantly predicts SD, positively, and ST, negatively, while GPC+PC and myo-inositol negatively predict ST. Although the correlation with TCI was only weak, age was used as covariate for ensuring to factor out any possible effect. In any case, the effect of a covariate on the results is proportional to the strength of its relation with the dependent variable. Since it has been reported that PCr+Cre plays a key role in brain energy homeostasis, increasing PCr+Cre levels may boost brain performance, as suggested by previous investigations in neurological (31) and healthy (32) conditions. Indeed, in these studies, PCr+Cre was found to have significant neuroprotective effects (31), and its biochemical levels have been shown to be involved during mental training (32). Additionally, oral Cre supplementation has been reported to have significant effects during calculation, in particular reducing mental fatigue and oxygen demand during the task (33) as well as to have positive effects on working memory and intelligence (34), further supporting its role in dynamically modulating brain energy capacity during cognitive performance. Importantly, it has been demonstrated that SD dimension of the TCI showed important correlations with other personality models or questionnaires, including the five-factor model (FFM) (35) and the Big Five Questionnaire (BFQ) (36). Specifically, it has been reported that SD directly correlates with conscientiousness and extraversion, and inversely, with neuroticism, dimensions of the FFM, as well as positively correlated with conscientiousness, emotional stability, and dynamism dimensions of the BFQ (36) in healthy adult subjects. Therefore, the neuroimaging studies investigating the putative association between these FFM and BFG dimensions and brain deficits might be useful to further support our results. Indeed, one resting-state fMRI study reported that conscientiousness and extraversion predicted resting state functional connectivity in several brain areas, including the anterior cingulate cortex in adult subjects (37). Moreover, a structural MRI study also showed that prefrontal volumes were larger in adults with higher conscientiousness and smaller in those with higher neuroticism (38). Therefore, all together, these results seem to point toward the hypothesis that deficits in prefrontal regions including the anterior cingulate cortex, might be associated with specific personality traits.

Finally, our results also showed a negative association of GPC+PC and myo-inositol in the anterior cingulate cortex with ST in our group of adolescents. Also, in this case, our result is not surprising especially because it has been reported that anterior cingulate cortex is involved in decision making and deployment of cognitive control (39) as well as in carefulness, industriousness, and organization activities (37), which might not be directly associated with ST traits. Indeed, higher scores in ST might identify subjects with higher levels of creativity, spirituality, and mysticism (7), all activities that do not require the engagement of higher order cognitive regions such as the anterior cingulate cortex, ultimately suggesting that this region needs a lower metabolic availability in individuals who rank high in ST.

### Limitations

Our findings should be considered in light of some methodological limitations, which could have potentially affected the reliability and generalizability of our results. First, the technique that we employed does not assess the brain biochemistry at whole brain level, but only within a restricted region of interest. This constrains the results’ generalizability and the comparability to previous structural personality studies.

Secondly, each metabolite was entered as predictor, one at a time, in discrete simple regression models, instead of running comprehensive multiple regressions (i.e., including all the metabolites as independent variables contributing to the TCI’s data distributions). The choice was mandatory because of the collinearity between the independent variables, most of which were correlating between each other. This grew the number of models that were tested, so increasing the risk for false discovery. Nonetheless, this risk was overcome by correcting the significance level for multiple comparisons (see paragraph 2.5 for details). Third, although the sample was relatively small, it was well selected for the absence of potentially confounding variables. Despite these limitations, the data were reasonably distributed across the sample and the conclusions were not driven by unbalances between the participants.

### Conclusions

Despite these limitations, our results may represent a step forward for personality studies, as we began to discriminate the biochemical systems underpinning personality functioning. Furthermore, understanding the biological mechanisms building personality traits may provide new insight on the mechanisms of drug action, which may ultimately lead to more rapid and effective treatments of personality disorders. Indeed, since it has been also reported that frontal lobe PCr+Cre is a valid treatment target in adolescent depression (20) and SD has been suggested to be a general trait marker for depression (21, 22), it is plausible that interventions aiming at modulating PCr+Cre concentrations might be a new effective strategy for the treatment of either dysfunctional personality traits or depression. This is because it has been demonstrated that, even in healthy brains (19), increasing creatine levels following oral creatine supplementation modify brain energy metabolism, which has been found to be altered in various psychiatric illnesses, including depression and schizophrenia (19). Therefore, future ^1^H MRS studies are warranted to further explore the role of brain chemistry on major psychiatric disorders. Finally, overall, our results suggest not only that personality traits are associated with specific biochemical circuits but also that this association is present already during adolescence, ultimately underlining the importance of investigating the relationship between personality traits and biological measures during the development. However, future larger studies are needed to better discriminate the biomarkers characterizing the different personality traits.

## Data Availability Statement

The datasets generated for this study are available on request to the corresponding author.

## Ethics Statement

The studies involving human participants were reviewed and approved by Ethical committee of the IRCCS “E.Medea.” Written informed consent to participate in this study was provided by the participants’ legal guardian/next of kin.

## Author Contributions

PB designed the study. LS and JS performed the MR analyses. GD, CaB, LS, JS, and PB performed the statistics. MN and PB supervised enrolment and evaluation of the subject sample. MMa, CaB, MG, and SP recruited and administered scales and interviews. MMa, BT, CaB, and FF contributed to data collection and eased accessibility to MR facilities. LS, CaB, and PB wrote the first version of the manuscript. GD and JS revised the earlier versions of the manuscript. All authors contributed to the writing and accepted the final version of the manuscript.
