# Corrigendum: The Assertive Brain: Anterior Cingulate Phosphocreatine Plus Creatine Levels Correlate With Self-Directedness in Healthy Adolescents

**DOI:** 10.3389/fpsyt.2019.00907

**Published:** 2020-01-09

**Authors:** Letizia Squarcina, Giuseppe Delvecchio, Maria Nobile, Maddalena Mauri, Domenico Madonna, Carolina Bonivento, Marco Garzitto, Sara Piccin, Massimo Molteni, Barbara Tomasino, Cinzia Bressi, Franco Fabbro, Jeffrey A. Stanley, Paolo Brambilla

**Affiliations:** ^1^ Department of Neurosciences and Mental Health, Foundation IRCCS Ca' Granda Ospedale Maggiore Policlinico, Milan, Italy; ^2^ Department of Pathophysiology and Transplantation, University of Milan, Milan, Italy; ^3^ Child Psychopathology Unit, Scientific Institute, IRCCS Eugenio Medea, Bosisio Parini, Italy; ^4^ School of Medicine and Surgery, University of Milano-Bicocca, Milan, Italy; ^5^ Scientific Institute, IRCCS Eugenio Medea, San Vito al Tagliamento, Italy; ^6^ Department of Psychiatry and Behavioral Neurosciences, School of Medicine, Wayne State University, Detroit, MI, United States

**Keywords:** magnetic resonance spectroscopy, temperament character inventory, adolescence, brain biochemistry, brain metabolism

In the original article, there was an error. The number of males and females was incorrectly listed in the Abstract as “9 males and 18 females” instead of “17 males and 9 females”.

A correction has been made to the **Abstract**:

“Despite various advances in the study of the neurobiological underpinnings of personality traits, the specific neural correlates associated with character and temperament traits are not yet fully understood. Therefore, this study aims to fill this gap by exploring the biochemical basis of personality, explored with the temperament and character inventory (TCI), during brain development in a sample of adolescents. Twenty-six healthy adolescents (aged between 13 and 21 years; 17 males and 9 females) with behavioral and emotional problems underwent a TCI evaluation and a 3T single-voxel proton magnetic resonance spectroscopy (¹H MRS) acquisition of the anterior cingulate cortex (ACC). Absolute metabolite levels were estimated using LCModel: significant correlations between metabolite levels and selective TCI scales were identified. Specifically, phosphocreatine plus creatine (PCr+Cre) significantly correlated with self-directedness, positively, and with a self-transcendence (ST), negatively, while glycerophosphocholine plus phosphocholine (GPC+PC) and myo-inositol negatively correlated with ST. To the best of our knowledge, this is the first study reporting associations of brain metabolites with personality traits in adolescents. Therefore, our results represent a step forward for personality neuroscience within the study of biochemical systems and brain structures.”

The authors apologize for this error and state that this does not change the scientific conclusions of the article in any way. The original article has been updated.

