# Supplementary material for: The Assertive Brain: Anterior Cingulate Phosphocreatine plus Creatine Levels Correlate With Self-Directedness in Healthy Adolescents
Source: Front Psychiatry. 2019 Nov 5;10:763. doi: 10.3389/fpsyt.2019.00763 (PMC6849467; doi:10.3389/fpsyt.2019.00763)
Supplement: Supplementary file 1 [file DataSheet_1.docx]

Supplementary Material

# Supplementary Figures


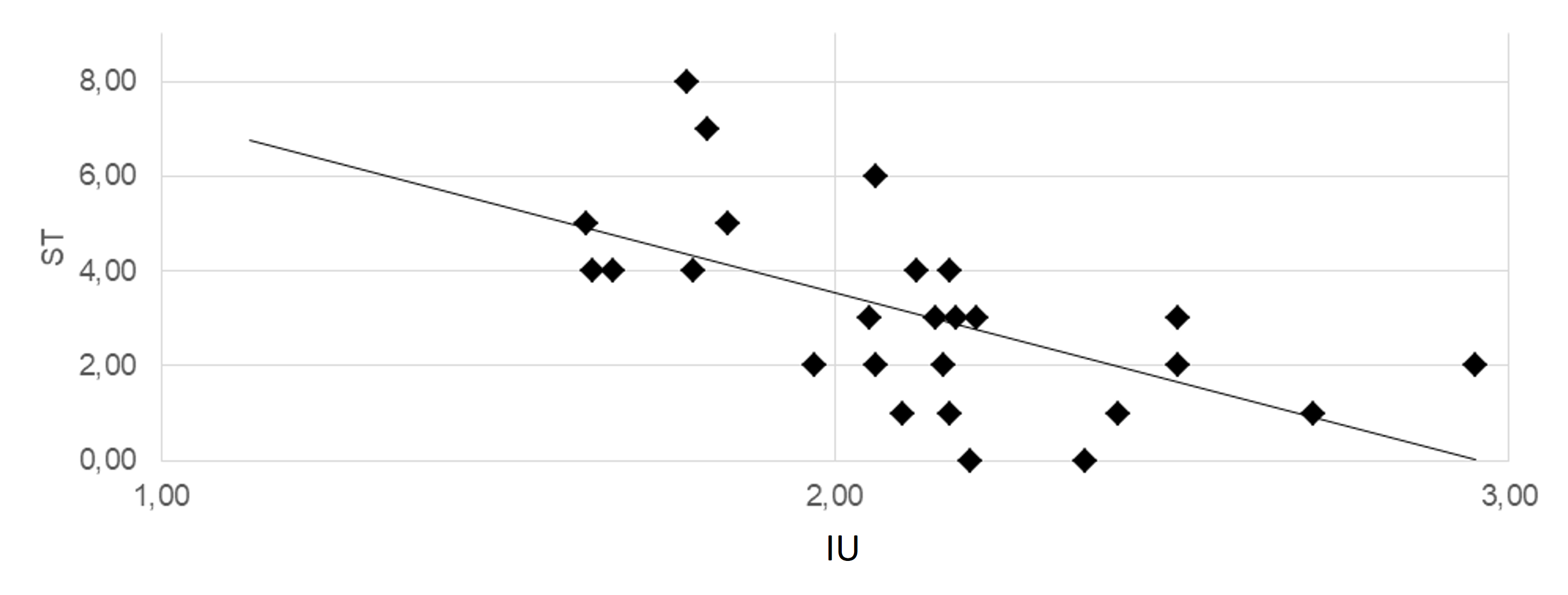


**Supplementary Figure S1.** Scatter plot depicting the relationship of GPC+PC concentration and TCI Self-transcendence scores. The solid line represents the linear regression line. The correlation coefficient r is -0.60 (Pearson correlation, p = 0.001). IU = institutional units.


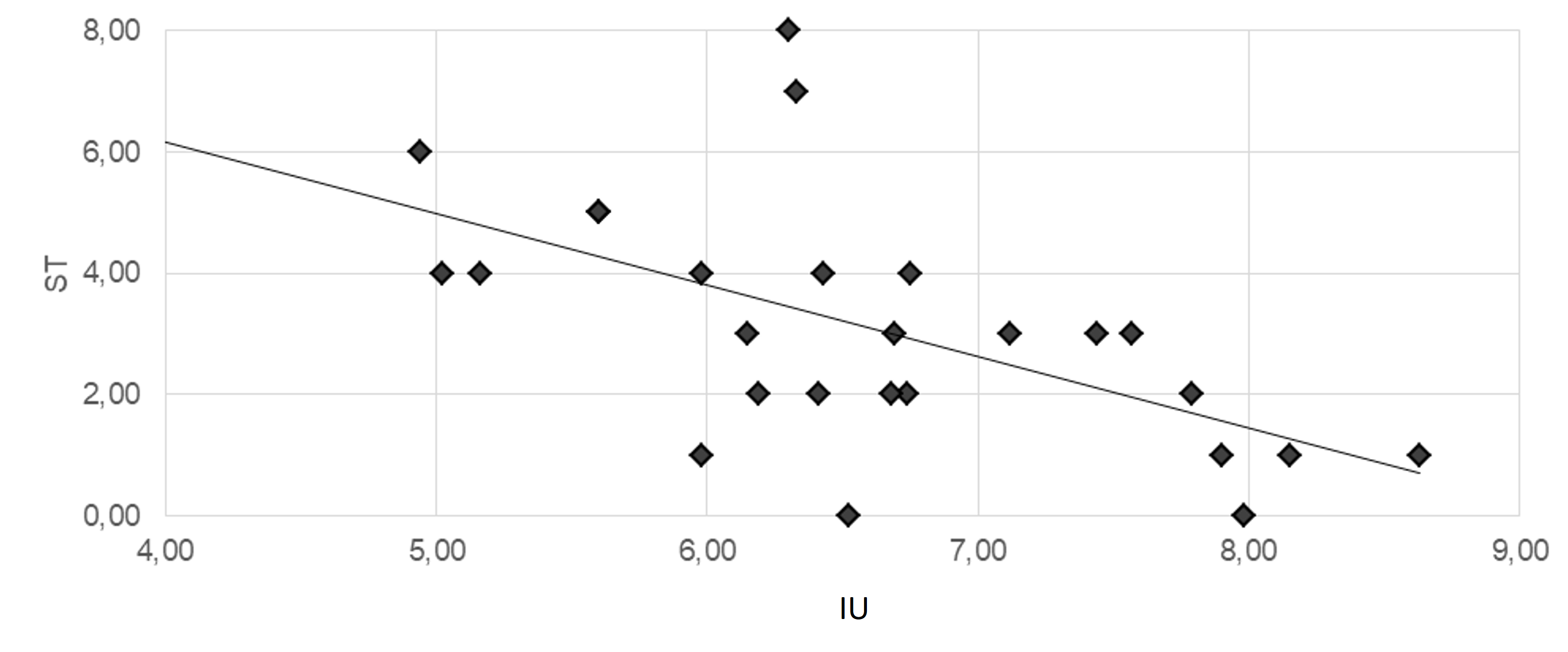


**Supplementary Figure S2**. Scatter plot depicting the relationship of myo-inositol concentration and TCI Self-transcendence scores. The solid line represents the linear regression line. The correlation coefficient r is -0.57 (Pearson correlation, p = 0.002). IU = institutional units.

**Supplementary Table S1.** Correlation between TCI scores and those covariates

|  | **TCI_NS**  **(p)** | **TCI_HA**  **(p)** | **TCI_RD**  **(p)** | **TCI_P**  **(p)** | **TCI_SD**  **(p)** | **TCI_CO**  **(p)** | **TCI_ST**  **(p)** |
| --- | --- | --- | --- | --- | --- | --- | --- |
| **CBCL_INT** | 0.074  (0.721) | **0.441**  **(0.024)** | -0.361  (0.07)0 | -0.224  (0.271) | **-0.570**  **(0.002)** | -0.212  (0.297) | 0.278  (0.169) |
| **CBCL_EXT** | **0.491**  **(0.011)** | 0.161  (0.431) | -0.128  (0.533) | -0.042  (0.839) | **-0.483**  **(0.012)** | -0.169  (0.408) | 0.284  (0.159) |

CBCL = child behavior checklist; TCI = Temperament and Character Inventory; Int = Internalizing Problems Index; Ext = Externalizing problems index; SD = self-directedness; ST = self-transcendence; CO = cooperativeness; NS = novelty seeking; HA = harm avoidance; RD = reward dependency; P = persistence. Reported correlation values are Pearson’s correlation coefficients.

**Supplementary Table S2.** Normal, Clinical and Subclinical subjects, based on Internalizing or Externalizing problems.

|  | **DAWBA INT** | **DAWBA EXT** |
| --- | --- | --- |
| **Normal** | 16 | 22 |
| **Clinical** | 4 | 1 |
| **Subclinical** | 6 | 3 |

DAWBA = Development and Well-Being Assessment; Int = Internalizing Problems Index; Ext = Externalizing problems index;
